# A London Holiday for Hospital Men

**Published:** 1917-06-30

**Authors:** 


### A LONDON HOLIDAY FOR HOSPITAL MEN.

The London hospital staffs are now so short-handed and so busy that holidays will be unusually hard to arrange, apart from the anxiety and emergency which may be at any moment created for them by the repetition of an air raid. For this reason those who are unable to get away as usual, and they will be many, may well be grateful for any hints as to how to spend a holiday in town. We have two suggestions to offer, and any others which our correspondents may have to make are likely to be welcome.

It is the essence of a London holiday for those who are Londoners that it should involve no change of address, but be itself a change which can be enjoyed at leisure moments without carrying the traveller fast or far afield. It therefore resolves itself into exploring things at hand which are usually forgotten, or turning attention to places and people which a man does not ordinarily meet. Since, however, few people can bring themselves unaided to hunt out the curious or the beautiful in their native city, some organisation which shall arrange details is required. It may be provided, among others, by the Selborne Society, which, as many readers know, provides "rambles" and guides to most of the interesting places in the London area and the Home Counties. These are open, we understand, to the general public as well as to members. About four "rambles" are arranged every month, and, by way of example, we may mention some of the forthcoming expeditions. On June 22 and Saturday, June 23, a "Nature in London" Exhibition was held at the Ashburton Club, 28 Red Lion Square, W.C. 1. Plant, tree, and animal life in London form the subjects of the exhibits, illustrated by photographs and specimens, while the open spaces of London have a department to themselves. Information can be obtained either from the Selborne Society, Avenue Chambers, Bloomsbury Square, London, W.C., or from the London Country Holidays Association. Among the Selborne Society's forthcoming "rambles" is an evening one through the Italian quarter, another on Saturday, June 30, to Kew Gardens, under the guidance of Professor G. S. Boulger, and a third on Saturday, July 7, to Twickenham. During July afternoon and evening rambles have been arranged to the William Penn country—that is to say, Jordans and Chalfont St. Giles, while "America in London" and "London through the Eyes of Benjamin Franklin" form the subject of two other expeditions. Mr. W. H. Daun, 155 Fenchurch Street, E.C., one of the Society's excursion secretaries, will provide any information desired. Those who remember the Society's rambles through "Old Chelsea," and other interesting bits of Old London, know how delightful these rambles may be with a good guide who knows how to plan them and to show everything worth seeing.

Another form of holiday-making for those who prefer the excitement of lectures and the stimulus of debate will be provided by the Summer Meeting, which is to be held at the Hampstead Garden Suburb from August 3 to August 17, when many well-known speakers are to open discussions or deliver lectures on the various "Problems of Reconstruction After the War." The Institute, Central Square, is to be the centre of Mrs. Barnett's meetings. Those who cannot leave London have a certain chance here of stimulus and enjoyment under pleasant surroundings and skilled leadership. International Relations, Ideals of Education, and Arts and Crafts are among the chief sections into which the conferences will be divided. The speakers include many well-known names, of which only a few can here be mentioned. Sir Oliver Lodge, the Master of Balliol, Mr. Raymond Unwin, Mr. Graham Wallas, Professor John Adams, the Hon. Edward Lyttelton, Miss Mary Macarthur, Professor Selwyn Image, Mr. George Clausen, R.A., and Mr. J. A. Spender are among the speakers. Certain excursions have also been arranged, and the tickets for the meeting, excluding these, can be obtained, price one guinea, from Miss Bolden, at the Institute, Hampstead Garden Suburb, N.W. 4. It should be added that Lord Leverhulme has kindly thrown open his picture gallery and collection of porcelain to members whom he has invited to an afternoon party at his Hampstead house. For those who are able to spend a day or two away accommodation is available, and addresses can be furnished, though the Tube has made Golders Green accessible to all. These two suggestions are sufficient to show that busy people who cannot leave town have more ways than one of spending a holiday in London.

